# Time-lapse observation of stepwise regression of Erk activity in zebrafish presomitic mesoderm

**DOI:** 10.1038/s41598-018-22619-9

**Published:** 2018-03-12

**Authors:** Dini Wahyu Kartika Sari, Ryutaro Akiyama, Honda Naoki, Hannosuke Ishijima, Yasumasa Bessho, Takaaki Matsui

**Affiliations:** 1 Gene Regulation Research, Nara Institute of Science and Technology, 8916-5 Takayama, Nara, 630-0101 Japan; 2 Department of Fisheries, Faculty of Agriculture, Universitas Gadjah Mada, Jl. Flora, Bulaksumur, Yogyakarta, 55281 Indonesia; 3 Laboratory of Theoretical Biology, Graduate School of Biostudies, Kyoto University, Yoshidakonoecho, Sakyo, Kyoto, 606-8315 Japan

## Abstract

During somite segmentation, clock genes oscillate within the posterior presomitic mesoderm (PSM). The temporal information ties up with the posteriorly moving FGF gradient, leading to the formation of a presumptive somite within the PSM. We previously investigated Erk activity downstream of FGF signaling by collecting stained zebrafish embryos, and discovered that the steep gradient of Erk activity was generated in the PSM, and the Erk activity border regularly shifted in a stepwise manner. However, since these interpretations come from static analyses, we needed to firmly confirm them by applying an analysis that has higher spatiotemporal resolutions. Here we developed a live imaging system for Erk activity in zebrafish embryos, using a Förster resonance energy transfer (FRET)-based Erk biosensor. With this system, we firmly showed that Erk activity exhibits stepwise regression within the PSM. Although our static analyses could not detect the stepwise pattern of Erk activity in clock-deficient embryos, our system revealed that, in clock-deficient embryos, the stepwise regression of Erk activity occurs at an irregular timing, eventually leading to formation of irregularly-sized somites. Therefore, our system overcame the limitation of static analyses and revealed that clock-dependent spatiotemporal regulation of Erk is required for proper somitogenesis in zebrafish.

## Introduction

The metameric structures of the vertebrate body, including the axial skeleton and skeletal muscle, are derived from a linear array of embryonic segments called somites^1–4^. The somites, which lie on each side of the embryonic midline, are generated by the periodic segmentation of the anterior end of the presomitic mesoderm (PSM). A “clock and wavefront” model was proposed in the 1970s to explain the periodic segmentation of somites^5^. In this model, the clock (a cellular oscillator) and the wavefront (a posteriorly moving front that arrests the clock, leading to rapid cell-fate changes) are present in the PSM. Through the identification of the cellular oscillators and wavefront in various vertebrates, such as the mouse, chick, and zebrafish^2,6^, this model has provided key insights on the process of somite segmentation^7–12^. The cellular oscillators include *Hes7* (a *Hes* family transcription factor) and *Lunatic fringe* (*Lfng*, a Notch effector) in the mouse^13,14^, and *her1* and *her7* (*Hes* family transcription factors) and *deltaC* (a Notch ligand) in the zebrafish^15,16^. Fibroblast growth factor (FGF) and Wnt signaling produce gradients that weaken at the anterior end, and the gradients move continuously toward the posterior end alongside tail elongation^2,17–20^. Because of changes in somite size induced by the inhibition and stimulation of FGF signaling, the wavefront represents the FGF threshold^2,20,21^.

To understand how segmental pre-patterns are determined within the PSM, we previously investigated Erk activity downstream of FGF signaling by collecting stained zebrafish embryos^21^. We discovered that the *fgf8a* gradient (wavefront) was converted into a step-like gradient of Erk activity with a sharp border in the PSM, and that the Erk activity border regularly shifted in a stepwise manner. However, the stepwise pattern of Erk activity could not be detected in clock-deficient embryos (*her1* and *her7* double morphants). These findings suggested that the Erk activity border regularly shifted by the clock (*her1* and *her7*) in a stepwise manner within the uniform PSM. However, since these interpretations come from static observations, whether stepwise regression of Erk activity occurs in the uniform PSM of living zebrafish embryos and what is the role of Erk stepwise regression need to be further investigated.

In this study, we used a Förster resonance energy transfer (FRET)-based Erk biosensor and succeeded to visualize Erk activity within living zebrafish embryos. Erk biosensor detected high Erk activity in the posterior PSM with a sharp border. Time-lapse FRET imaging revealed that the Erk activity border shifts stepwisely along with a regular timing in control embryos, but, in clock-deficient embryos, the stepwise regression of Erk activity occurs at irregular timing. Furthermore, we found a positive correlation between the timing and corresponding somite size in clock-deficient embryos, suggesting that Erk stepwise regression with a regular timing is required for cyclic formation of normal sized somites. Therefore, our system succeeded to visualize Erk activity in living zebrafish embryos and revealed a novel spatiotemporal regulation of Erk in the PSM required for proper formation of somites in zebrafish.

## Results

### The visualization of the segmental pre-pattern in living zebrafish embryos

**Figure 1:**
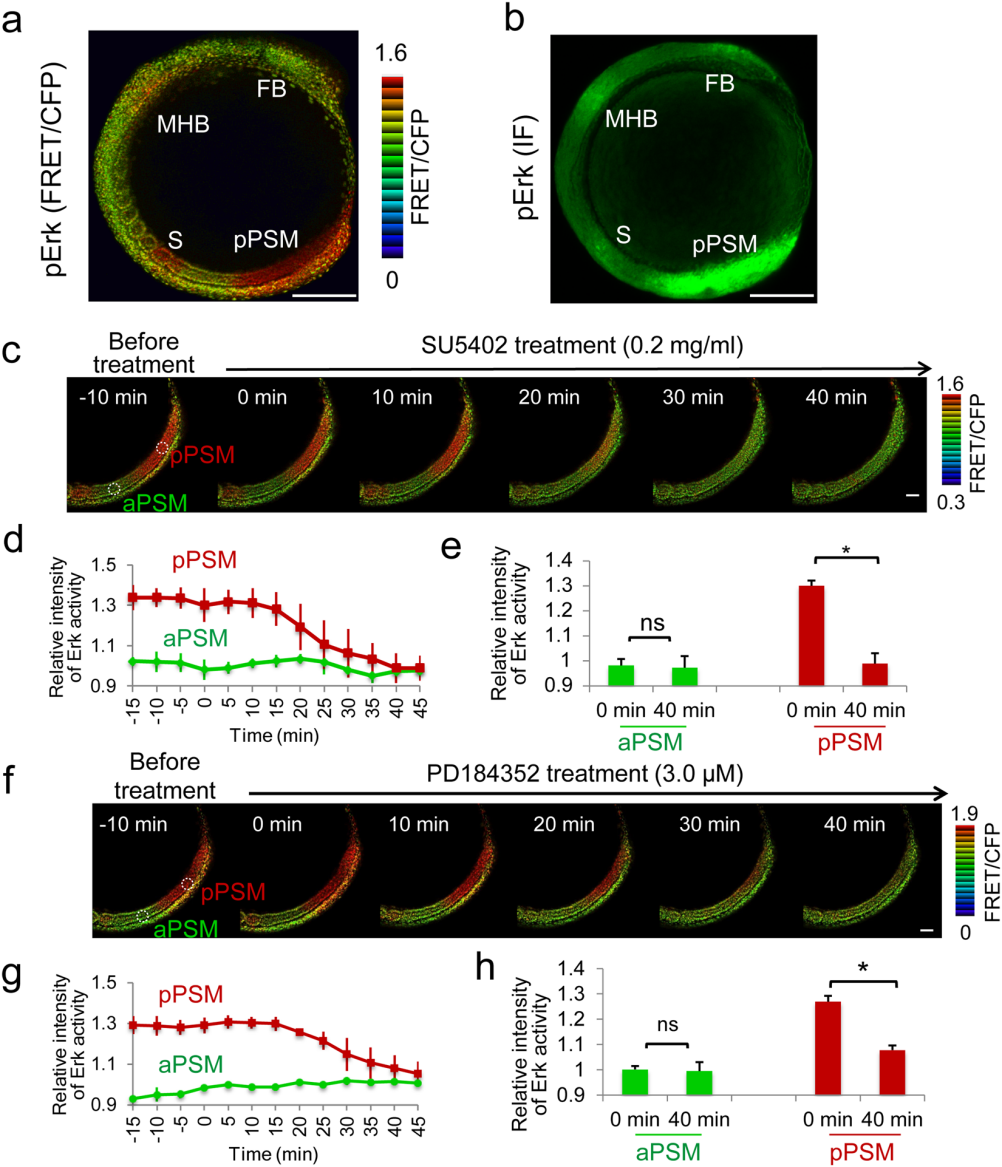
Erk biosensor successfully monitors FGF/Erk signaling in zebrafish embryos. **(a)** Erk activity detected by the Erk biosensor in control (8-color heat map) (upper panel). **(b)** Distribution of pErk detected by an anti-pErk antibody (green). Lateral view, anterior to the top. FB, forebrain; MHB, midbrain–hindbrain boundary; S, somite I; and pPSM, posterior PSM. Scale bar, 200 μm. IF, immunefluorescent staining. **(c–h)** A FGFR inhibitor (0.2 mg/ml SU5402) **(c–e)** and a MEK inhibitor (3.0 μM PD184352) **(f–h)** blocked Erk activity as detected by the Erk biosensor in zebrafish embryos (also see Movies 1 and 2). Still images from original movies are shown **(c,f)**. Scale bar, 50 μm. Signal intensity was measured within a white circle (panels c, f) positioned in the anterior PSM (aPSM, as a negative region) or posterior PSM. SU5402 (n = 3) and PD194352 (n = 5) treatment did not affect the signal intensity in the anterior PSM (green, in panels c, d, f, g). However, inhibitor treatment gradually inhibited the signals in the posterior PSM (red, in panels c, d, f, g). Signal intensity was not change in the anterior PSM between 0 and 40 min after inhibitor treatment (**e**,**h**), but a statistically significant difference (*P < 0.01) was seen in the posterior PSM between 0 and 40 min after inhibitor treatment (**e**,**h**). Error bars, standard deviations. ns, not significant.

Somite segmentation and segmental pre-pattern formation are dynamic processes during development. Because of the activation of Erk by FGF in newly-formed somites (somites I and II: SI and SII), which form from the somite (somite 0: S0), and posterior PSM regions^20,21^, and due to the relationship between the sharp border in Erk activity and the segmental pre-pattern at the posterior PSM^21^, we visualized the dynamic changes in Erk activity in living zebrafish embryos using a FRET-based Erk biosensor^22^. *Erk biosensor* mRNAs were injected into zebrafish embryos, and Erk activity was calculated using the FRET/CFP ratio in embryos at the 8-somite stage (8SS). Although the Erk biosensor was delivered into the entire body of the embryo, high FRET/CFP signals were detected in specific regions of the embryos, such as in the forebrain, SII, SI, S0, and posterior PSM, as well as at the midbrain–hindbrain boundary (Fig. 1a). In addition, its pattern was similar to that of phosphorylated Erk (pErk, the active form), which was detected with an anti-pErk (pErk) antibody^21^ (Fig. 1b). To determine whether the Erk biosensor can monitor Erk activity in living zebrafish embryos, we inhibited FGF/Erk signaling with a pharmacological inhibitor for FGFR1 or MEK. After treatment with either the FGFR1 inhibitor (SU5402) or the MEK inhibitor (PD184352), the high FRET/CFP signals in the posterior PSM of 8SS embryos gradually decreased to their basal levels (Fig. 1c–h and Movies 1 and 2), confirming that the FRET/CFP signals indeed reflect FGF-dependent Erk activity, and that the Erk biosensor can monitor Erk activity in living zebrafish embryos.

**Figure 2:**
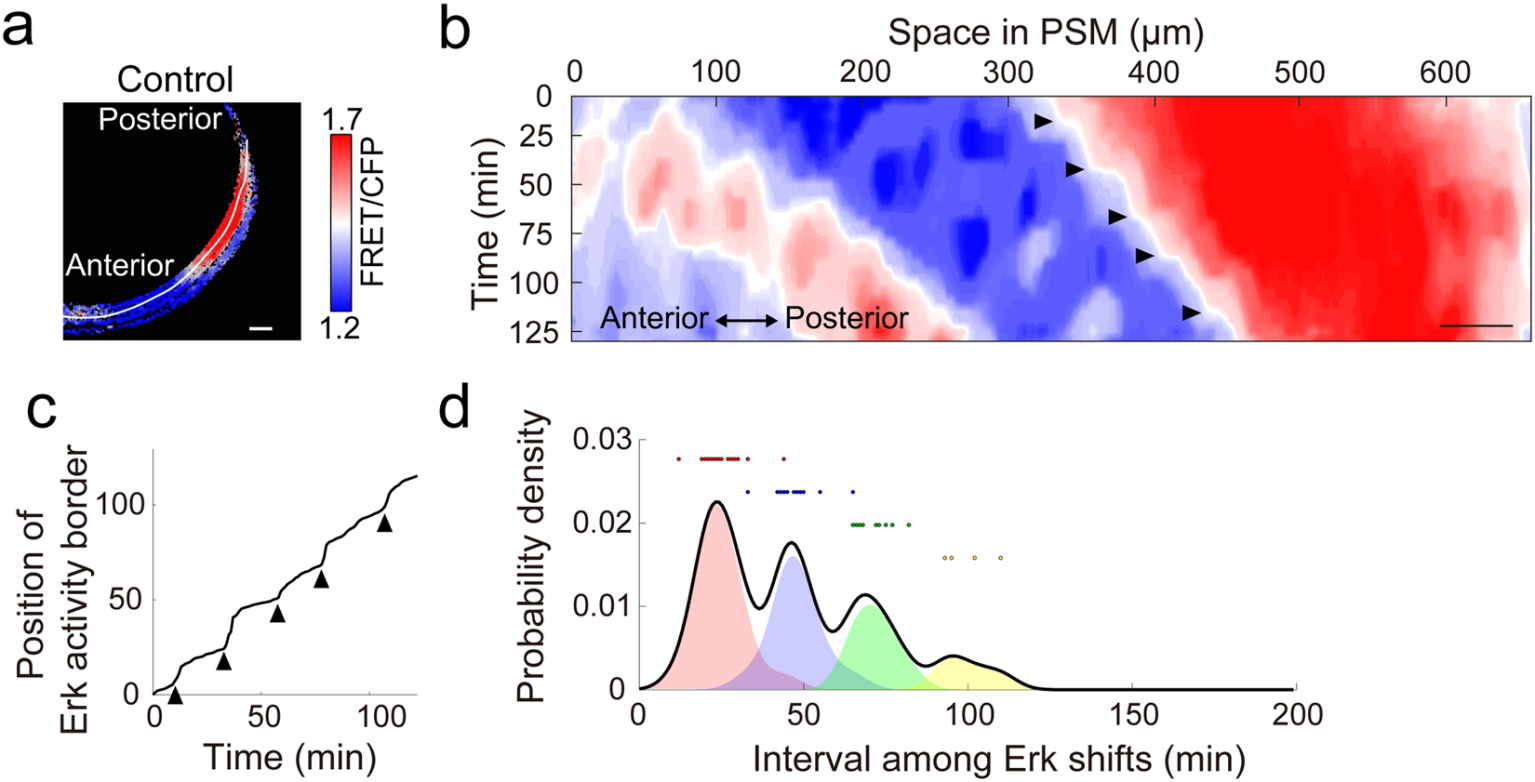
Stepwise regression of Erk activity in control embryos. **(a)** A line of interest for kymograph analysis in a control embryo (3-color heat map). Scale bar, 50 μm. **(b)** Kymograph of the control embryo. Horizontal and vertical axes indicate the length from the anterior end of the PSM (μm) and the progression of time (min), respectively. Scale bar, 50 μm. Signal intensities are shown by the heat map. The original movie is provided as Movie 3. **(c)** A contour corresponding to the Erk activity border was extracted from the kymograph (panel b). Arrowheads indicate the change-points of the curve and correspond to the time of the stepwise shifts of the Erk activity border. Time-dependent positional changes could be seen in the control embryo. **(d)** Distribution of time interval among the stepwise shifts of Erk activity. The distribution was generated from control embryos (n = 5). The black line indicates the distribution of time intervals for all stepwise shifts. Red, blue, green, and yellow shaded regions correspond to the distribution of time intervals between the indicated and the next stepwise shifts, or the subsequent two, three, or more than four consecutive stepwise shifts, respectively. Red, blue, green and yellow dots indicate the data points.

By immunostaining with the anti-pErk antibody and quantitative analysis of the stained embryos, we recently showed that pErk forms a sharp border in the posterior PSM, and that the sharp border displays stepwise shifts that strongly correlate with somite segmentation, suggesting that the border of Erk activity represents a segmental pre-pattern in the posterior PSM (a determination front in the clock and wavefront model)^21^. In agreement with this notion, we could observe sharp border of Erk activity in the posterior PSM by using Erk biosensor (Fig. 2a and Supplementary Fig. S1). We next investigated the temporal changes in Erk activity by time-lapse FRET imaging (Fig. 2 and Movie 3). We generated a kymograph along the PSM and extracted a contour of the FRET/CFP ratio, which corresponded to the position of the Erk activity border (Fig. 2b and c, and see Methods). We found that the Erk activity border shifted toward the posterior end in a stepwise manner (Fig. 2b and c). To evaluate its periodicity, we computed the change-points of the contour at which the curve of the contour changes suddenly (arrowheads in Fig. 2c) and investigated the distribution of the intervals using all combinations of the change-points (Fig. 2d, and see Methods). The distribution showed a regular periodic pattern, with a periodicity that was consistent with that of the segmentation cycle (Fig. 2d). Thus, the time-lapse readouts by Erk biosensor firmly showed that stepwise regression of Erk activity occurs in the PSM of zebrafish embryos.

**Figure 3:**
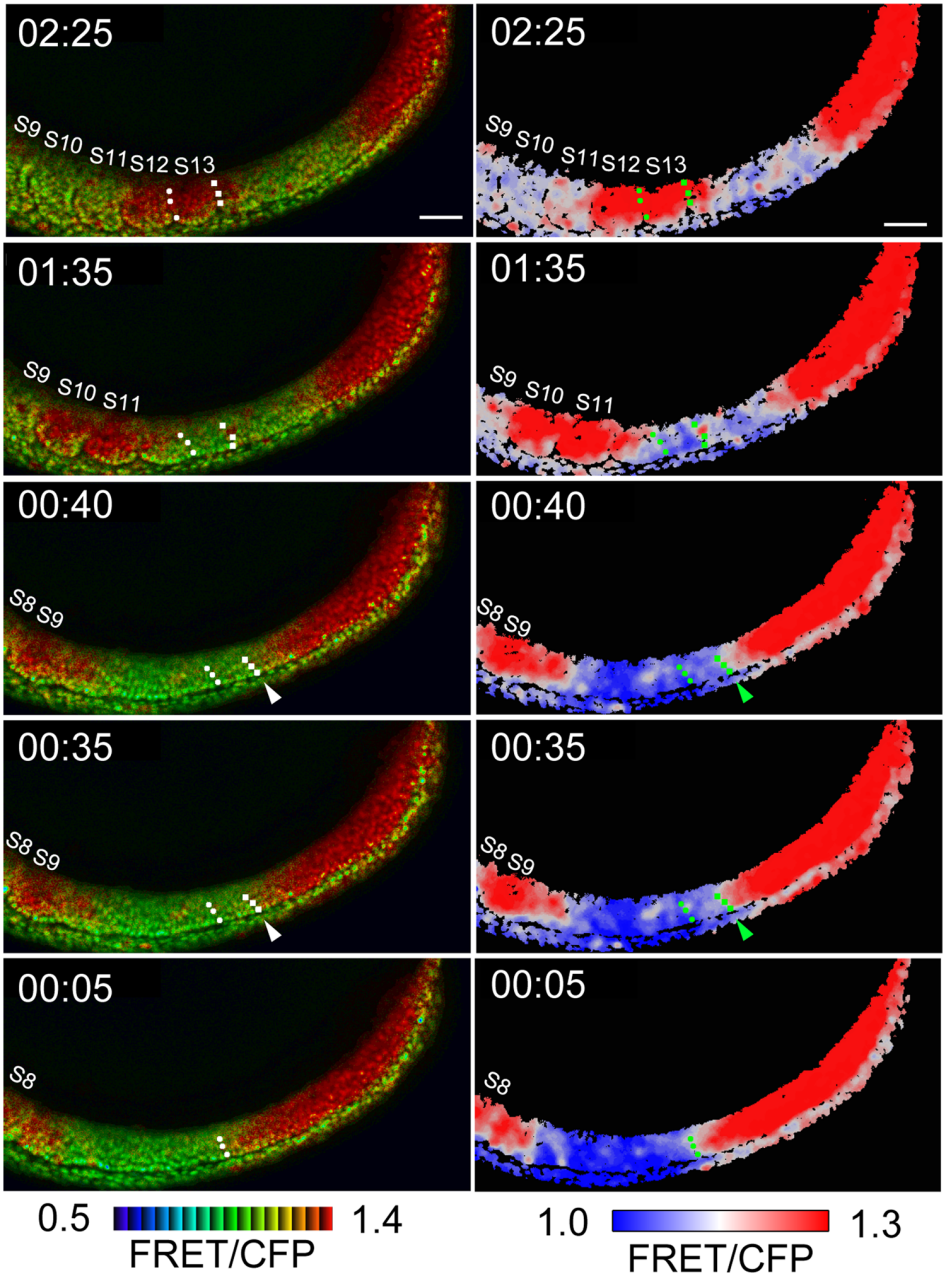
Retrograde cell tracking in control embryo. Left panel, 8-color heat map; right panel, 3-color heat map. Scale bar, 50 μm. At time point 02:25, anterior boundary cells of the S13 and forming somite (it will be the S14) were marked by dots (white in left panels; green in right panels), and their trajectories were retrogradely tracked. When time went back at time point 00:40, boundary cells in the forming somite (arrows) reached to Erk activity border, marked by central values of the FRET/CFP ratio, and this overlap was maintained at least for 5 min. The 5-min overlapping of the tracked cells with the Erk activity border could be seen in the trajectories of boundary cells of the S13 at time point 00:10-00:05. These results showed that Erk activity border corresponds to the segmental pre-pattern. The original movie is provided as Movie 4.

Based on our static analyses, we assumed that the sharp border of Erk activity represents the segmental pre-pattern within the uniform PSM^21^. To confirm this assumption, we performed retrograde tracking of multiple cells residing in the somite boundary. With this assay, we found that boundary cells of the somite were positioned with similar distance throughout the retrograde time-lapse observations, and that they were originated from the border of Erk activity within the PSM (Fig. 3 and Movie 4). These results firmly show that the sharp border of Erk activity corresponds to the segmental pre-pattern.

### The segmentation clock is not required for the formation and stepwise shift of the segmental pre-pattern in the posterior PSM

Clock-deficient embryos in the mouse (*Hes7*-deficient) and zebrafish (*her1* and *her7* double morphants and/or mutants) displayed severe skeletal and segmental defects^13,23^. In these mutants, however, somites are still formed, although the somite size was variable^13,21,23^. These findings suggest that the clock controls the regularity of the somite size rather than the formation of somites, and that a clock-independent mechanism, which spontaneously generates irregularly-sized somites, is embedded within somitogenesis. Currently, it is unclear how irregular somites form in clock-deficient embryos. To answer this question, we monitored the dynamic changes in Erk activity in embryos with a disrupted segmentation clock. The segmentation clock was disrupted by knocking down two clock genes, *her1* and *her7*^21,23^. In the manipulated embryos, Erk was activated in the forebrain, SI, and posterior PSM, as well as at the midbrain–hindbrain boundary (Supplementary Fig. S2a). In the posterior PSM, the sharp border of Erk activity was generated in a manner similar to that of control embryos (Supplementary Figs S1b and S2b), suggesting that the segmentation clock does not affect the formation of Erk activity border in the PSM.

**Figure 4:**
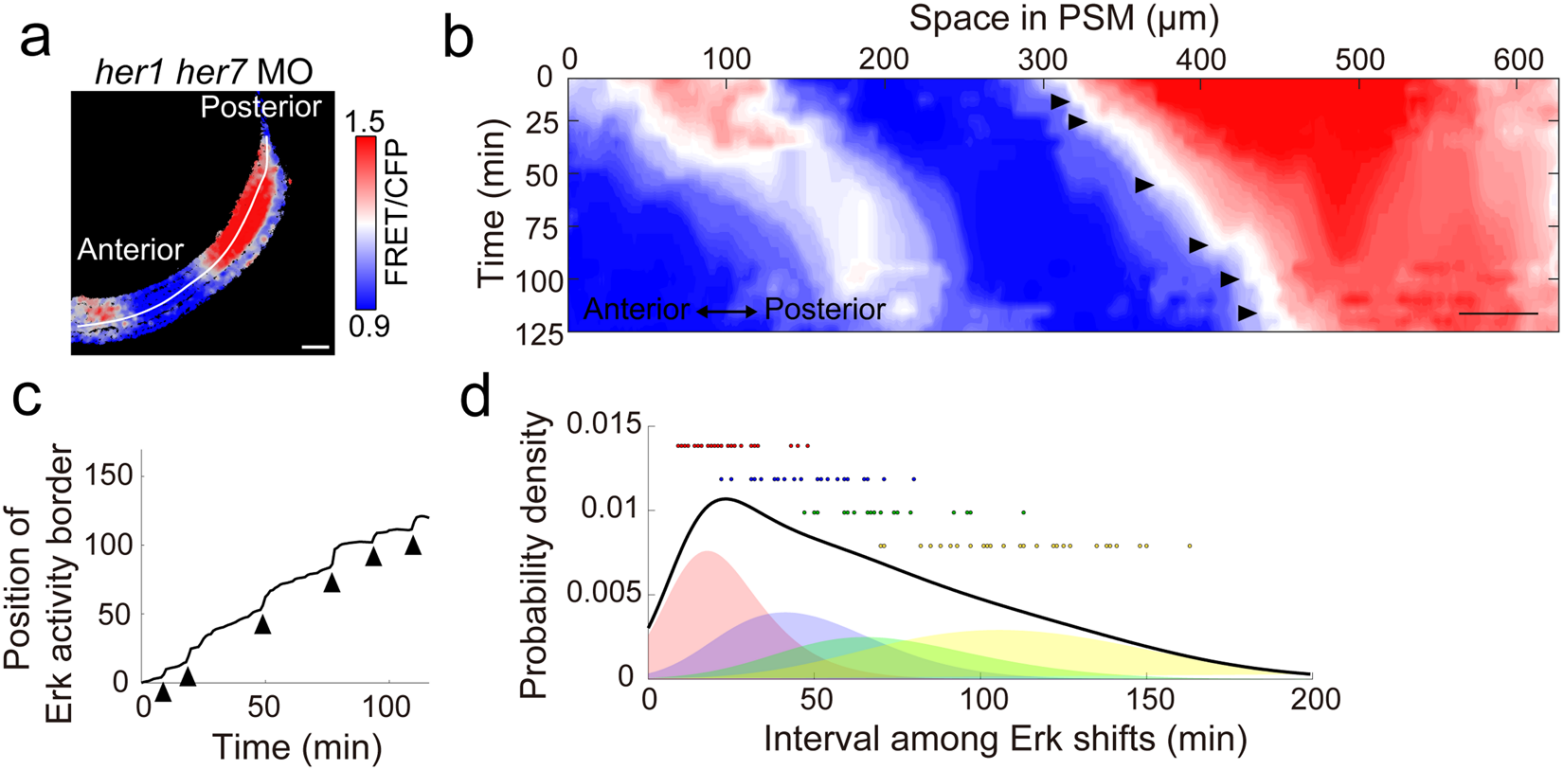
Stepwise regression of Erk activity in clock-deficient embryos. **(a)** A line of interest for kymograph analysis in a clock-deficient embryo (3-color heat map). Scale bar, 50 μm. **(b)** Kymograph of the clock-deficient embryo. Scale bar, 50 μm. The original movie is provided as Movie 5. **(c)** A contour corresponding to the Erk activity border was extracted from the kymograph (panel b). Arrowheads indicate the change-points of the curve and correspond to the time of the stepwise shifts of the Erk activity border. Time-dependent positional changes could be seen in the clock-deficient embryo. **(d)** Distribution of time intervals among the stepwise shifts of Erk activity. The distribution was generated from clock-deficient embryos (n = 5).

**Figure 5:**
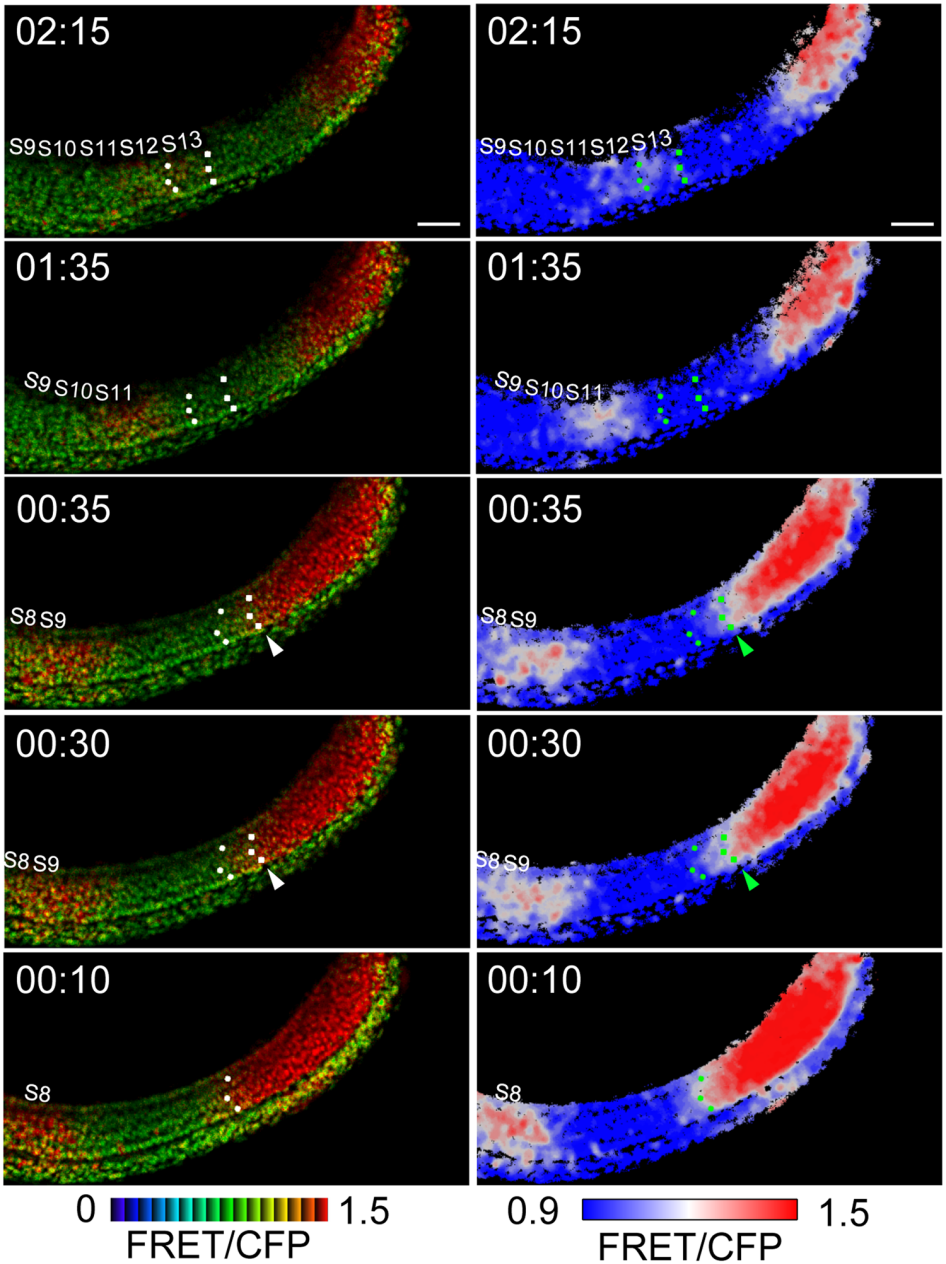
Erk activity border corresponds to the segmental pre-pattern even in clock-deficient embryos. Retrograde cell tracking in clock-deficient embryo was performed. At time point 02:15, anterior boundary cells of the S13 and forming somite (it will be the S14) were marked by dots (white in left panels; green in right panels), and their trajectories were retrogradely tracked. The 5-min overlapping of the tracked cells with the Erk activity border could be observed in the trajectories of boundary cells of somites (at time points 00:35-00:30 and 00:15-00:10). Left panel, 8-color heat map; right panel, 3-color heat map. Scale bar, 50 μm. The original movie is provided as Movie 6.

When we performed time-lapse FRET imaging and generated kymographs in clock-deficient embryos, we could find different dynamics of Erk activity border as compared to controls (compare Fig. 2 to Fig. 4): Erk activity border was generated in clock-deficient embryos as like controls, but the border shifted stepwisely with irregular timing (Fig. 4b–d). To test whether the sharp border of Erk activity could establish the segmental pre-pattern in the posterior PSM, even in clock-deficient embryos, we performed the retrograde tracking of multiple cells residing in the somite boundary in clock-deficient embryos. In agreement with data from control embryos (Fig. 3 and Movie 4), boundary cells residing in the somite were derived from the border of Erk activity within the PSM (Fig. 5 and Movie 6), suggesting that the border of Erk activity corresponds to the segmental pre-pattern, irrespective of the presence or absence of the segmentation clock.

### The segmentation clock may reduce the temporal irregularity of Erk stepwise regression, eventually leading to the formation of somites with a constant size

**Figure 6:**
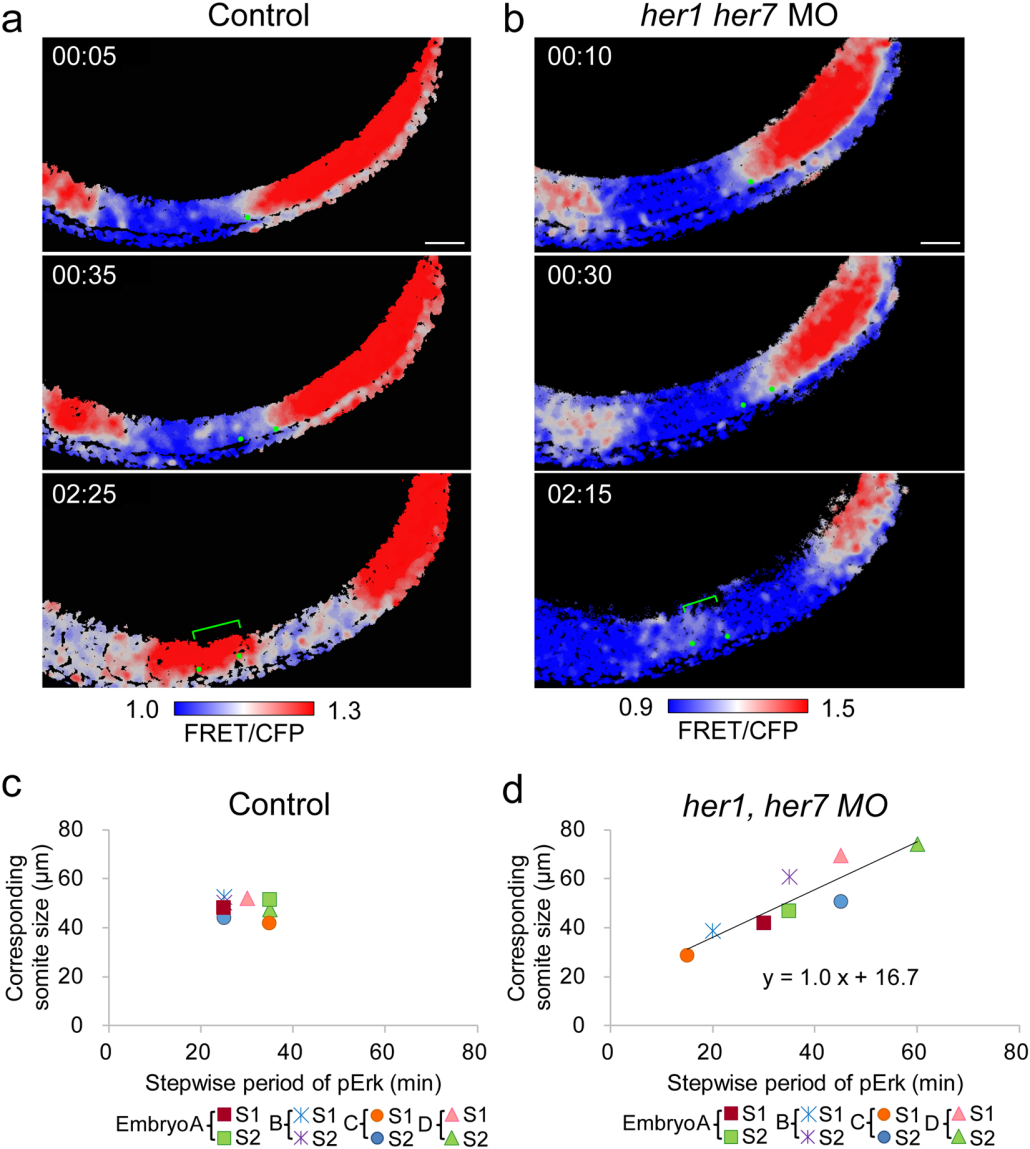
Correlation between the time interval between the sequential shift of Erk activity and the corresponding somite size. **(a)** Time intervals among the Erk stepwise shifts could be estimated from our time-lapse observations. In a control embryo (Embryo D in panel c), Erk activity borders appeared at 00:05 and 00:35. Thus, we considered the time interval among the sequential shift was 30 min. At the later time point (02:25), we could also estimate the size of the corresponding somite (52 µm), which is generated by the sequential stepwise cycle. Scale bar, 50 μm. The original movie is provided as Movie 7. **(b)** In a clock-deficient embryo (Embryo B in panel d), the time interval and corresponding somite size were 20 min and 39 µm, respectively. Scale bar, 50 μm. The original movie is provided as Movie 8. **(c,d)** In control (n = 4) and clock-deficient (n = 4) embryos, the time interval and corresponding somite size (total 8 somites) were measured and plotted. In control embryos, the time interval and corresponding somite size were constant **(c)**. However, in clock-deficient embryos, a positive linear correlation between them could be seen **(d)**.

Based on time-lapse observations in clock-deficient embryos (Fig. 4 and Movie 5), we assumed that the temporal irregularity of Erk stepwise regression reflects to the size variations of the corresponding somite. To test the possibility, we timed the interval between a sequential stepwise shift, and then measured the size of the corresponding somite, which is generated during the stepwise cycle (Fig. 6a,b). In control embryos, both the time interval of the sequential shift (29.4 ± 4.6 min, coefficient of variation (CV) = 0.16) and the corresponding somite size (48.5 ± 3.6 µm, CV = 0.07) were constant (Fig. 6c). However, in clock-deficient embryos, both the time interval (36.9 ± 12.5 min, CV = 0.34) and size (53.1 ± 13.9 µm, CV = 0.26) were highly variable as compared to controls. Although there were high variations in clock-deficient embryos, we were able to identify a positive linear correlation between them (Fig. 6d). For instance, when a time interval between the sequential stepwise shift was 20 or 45 min in clock-deficient embryos, the corresponding somite was smaller (39 μm) or larger (58 μm), respectively (Fig. 6d). This correlation suggests that the irregular somite formation in clock-deficient embryos depends on the temporal irregularity of stepwise shift of Erk activity. It also suggests a previously unidentified role for the segmentation clock in the posterior PSM: The segmentation clock reduces the temporal irregularity of the Erk stepwise regression and fine-tunes the corresponding somite size.

## Discussion

Our previous static analyses using anti-pErk antibody suggested that Erk activity is highly dynamic in the PSM during somitogenesis^21^. However, because the temporal resolution of static analyses was quite low, a live imaging system(s) that monitors Erk activity dynamics in the PSM is required. In this study, we introduced Erk biosensor into zebrafish embryos and could succeed to observe Erk activity dynamics in the PSM with a high resolution. In addition, we firmly confirmed the existence of a dynamic event that Erk activity shifts in a stepwise manner in the PSM, which is required for proper somitogeneis.

Our kymograph analyses suggest that central values of the FRET/CFP ratio, marked by white color in 3-color heat maps (e.g. Figs 2 and 4), correspond to Erk activity borders. This is supported by two lines of evidence. Firstly, the positions of these central values matched with the anterior extremities of Erk activity estimated from the signal intensity plot (Supplementary Fig. S1a), whose positions are also consistent with those of the segmental pre-patterns^21^. Secondly, retrograde tracking of multiple cells residing boundary cells of the somite revealed that these central values correspond to the segmental pre-pattern in the PSM (Figs 3 and 5). Taken together, we proposed that Erk activity borders, indicated by central values of the FRET/CFP ratio, show stepwise regression with constant or irregular timing in control or clock-deficient embryos, respectively (Figs 2 and 4, and Movies 3 and 5).

Our time-lapse readout suggests that proper formation of somites with a constant size is required for clock-dependent temporal regulation of Erk activity in the PSM. However, there is no direct evidence that the clock (*her1* and *her7*) temporally modulates Erk activity in the zebrafish, although the mRNA expression of negative Erk regulators, such as *sprouty* and *dusp*, oscillates in the mouse and chick^6^. Thus, for further investigation, it will be important to identify the functional relationship between the clock and Erk activity in the zebrafish, which may be different from that reported in the mouse and chick^6^.

The clock and wavefront model was proposed in 1976^5^, but was then revised after the molecular mechanism of somite segmentation was taken into account^24,25^. In addition to these models, several theoretical models have been described to explain the different aspects of somitogenesis, including the mechanism of oscillatory gene expression in PSM cells^9,26^, clock synchronization among cells^7,10–12^, and spatial oscillatory patterning in the PSM^8^. However, since the removal of the clock from these models predict an impedance of somite individualization, these models have never addressed how the spontaneous formation of irregular somites are formed in clock-deficient embryos. With our live imaging system, we could succeed to visualize Erk activity in the PSM of clock-deficient embryos, and found that Erk stepwise regression occurs with irregular timing in clock-deficient embryos, leading to the formation of irregularly-sized somites (Figs 4 and 6). To our best knowledge, this is the first study to monitor the molecular events of irregular somite formation in clock-deficient embryos. Our time-lapse readouts will be useful to generate theoretical models that can explain both irregular somite formation in the absence of the segmentation clock and normal somite formation in the presence of the segmentation clock.

## Methods

### Whole-mount immunohistochemistry

Whole-mount immunohistochemistry and *in situ* hybridization were performed as described previously^21,27,28^. An anti-pErk antibody (Sigma, cat. no. M8159) was used.

### Synthesis of mRNA encoding the Erk biosensor

The Erk biosensor Eevee-ERKnls comprises an enhanced cyan-emitting mutant of GFP (ECFP), a WW domain, an EV linker, an Erk substrate, a yellow fluorescent protein for energy transfer (Ypet), and a nuclear localization signal (NLS)^22^. When Erk phosphorylates the Erk substrate, the WW domain binds to the Erk substrate, thereby bringing ECFP closer to Ypet and inducing FRET from ECFP to Ypet. The pCS2-*Erk biosensor* subcloned from pPBbsr2-3594NLS^22^ was used as a template for mRNA synthesis. *Erk biosensor* mRNAs were synthesized using the SP6 mMessage mMachine system (Thermo Fisher Scientific).

### Morpholino oligonucleotides (MOs)

Control-MO (5′-CCTCTTACCTCAGTTACAATTTATA-3′), *her1*-MO (5′-TTCGACTTGCCATTTTTGGAGTAAC-3′), and *her7*-MO (5′-CAGTCTGTGCCAGGATTTTCATTGC-3′) were obtained from Gene Tools. Double knockdown of *her1* and *her7* using *her1*-MO and *her7*-MO phenocopies segmentation defects in the *b567* mutant, which is a deletion mutant of *her1* and *her7* genes^23^. The efficacy of these morpholino oligonucleotides (MOs) was also verified in our laboratory^21^.

### Preparation of zebrafish embryos for FRET imaging

*Erk biosensor* mRNA (100 pg), *her1*-MO (6.25 ng), and *her7*-MO (6.25 ng) were co-injected into the yolk of one-cell-stage zebrafish embryos as described previously^27^. As a control, *Erk biosensor* mRNA (100 pg) or *Erk biosensor* mRNA (100 pg) and *control*-MO (12.5 ng) were injected. Injected embryos were developed until the 7SS stage, dechorionated, and mounted laterally in 1% low melting point agarose at the indicated stages as described previously^29^.

### FRET microscopy

Embryos mounted in 1% agarose were observed under an LSM710 confocal microscope (Zeiss) equipped with a 440 nm diode laser and a Plan-Apochromat 10×/0.45 objective lens in the lambda scanning mode. Using the linear unmixing mode, CFP and Ypet signals were separated from the initial spectra data. Using MetaMorph software (Molecular Devices), the Ypet image was divided by the CFP image to generate FRET/CFP ratio images. The FRET/CFP ratio images were presented in the intensity-modulated display mode, which shows differences in the FRET/CFP ratio as either 8-color or 3-color heat map from red (high) to blue (low).

### Time-lapse FRET imaging

To determine whether the Erk biosensor could be used to monitor Erk activity in zebrafish embryos, 8SS embryos carrying the Erk biosensor were observed for 60 or 75 min at 5 min intervals at 28.5 °C. Between 15 and 20 min, the embryos were treated with SU5402 (0.2 μg/ml) or PD184352 (3.0 μM). To observe the dynamic changes in Erk activity in the PSM, *Z*-stack images (8 to 10 planes taken at 5 or 10 μm intervals) of 8SS embryos carrying the Erk biosensor were obtained at 5 min intervals at 28.5 °C.

### Image analysis

The FRET/CFP ratio images were generated with MetaMorph. The region of the sharp border of Erk activity was determined by comparing each FRET/CFP ratio image with the corresponding signal intensity plot. The positions of SII, SI, and S0 were determined by both visual observation (differential interference contrast) and Erk activation domains.

To confirm the stepwise shift of the borders of Erk activity in the posterior PSM, kymographs, which are graphical representations of the spatial position over time, were generated with MetaMorph following procedure. Time-lapse FRET/CFP ratio images were processed by median filter with 15 pixels windows. Line of interest on anterior-posterior axis in the PSM was selected to extract the signal intensity for kymograph. At each point along the line, Erk activities of vertically neighboring 16 pixels were averaged.

### Kymograph analysis

From kymograph images, we calculated contours, a set of isolines, by contour function in Matlab. Consequently, two-dimensional trajectories of isolines were also obtained as shown in Figs 2c and 4c. Among isolines around intermediate Erk activity region, the clearest stepwise trajectory was selected as the Erk activity border as shown in Figs 2c and 4c. For this end, velocity (first order derivative) of isoline trajectories were calculated in kymograph image space and then the one showing the largest variation of the velocity was selected. Note that for easy visualization of the Erk activity border, its activity level was set to white color in Figs 2b and 4b.

The change points of the Erk activity border, which correspond to sudden posterior shifts of its trajectory, were detected as follows. The negative second derivative of the Erk activity border velocity, which represents convexity, was calculated by Savitzky-Golay filter, and its peaks were selected as the change points.

### Retrograde cell tracking

By time-lapse FRET imaging in each embryo, we obtained a set of time series data as a Multi TIFF file. Using the “Reverse” function in ImageJ, we reversed time order of the Multi TIFF file. For cell tracking, we used the “Manual Tracking” plug-in in ImageJ. Multiple cells residing in the somite boundary were marked and tracked retrogradely.

### Statistical analysis

Differences between means were analyzed by one-tailed Student’s *t*-test. The results of *t*-tests were considered significant when *P* < 0.01.

## Electronic supplementary material

Supplementary information

Movie 1

Movie 2

Movie 3

Movie 4

Movie 5

Movie 6

Movie 7

Movie 8
