# Supplementary material for: Time-lapse observation of stepwise regression of Erk activity in zebrafish presomitic mesoderm
Source: Sci Rep. 2018 Mar 12;8:4335. doi: 10.1038/s41598-018-22619-9 (PMC5847612; doi:10.1038/s41598-018-22619-9)
Supplement: Supplementary file 1 — Supplementary information [file 41598_2018_22619_MOESM1_ESM.pdf]

Supplementary information

Time-lapse observation of stepwise regression of Erk activity in zebrafish presomitic mesoderm

Dini Wahyu Kartika Sari, Ryutaro Akiyama, Honda Naoki, Hannosuke Ishijima, Yasumasa Bessho, and Takaaki Matsui

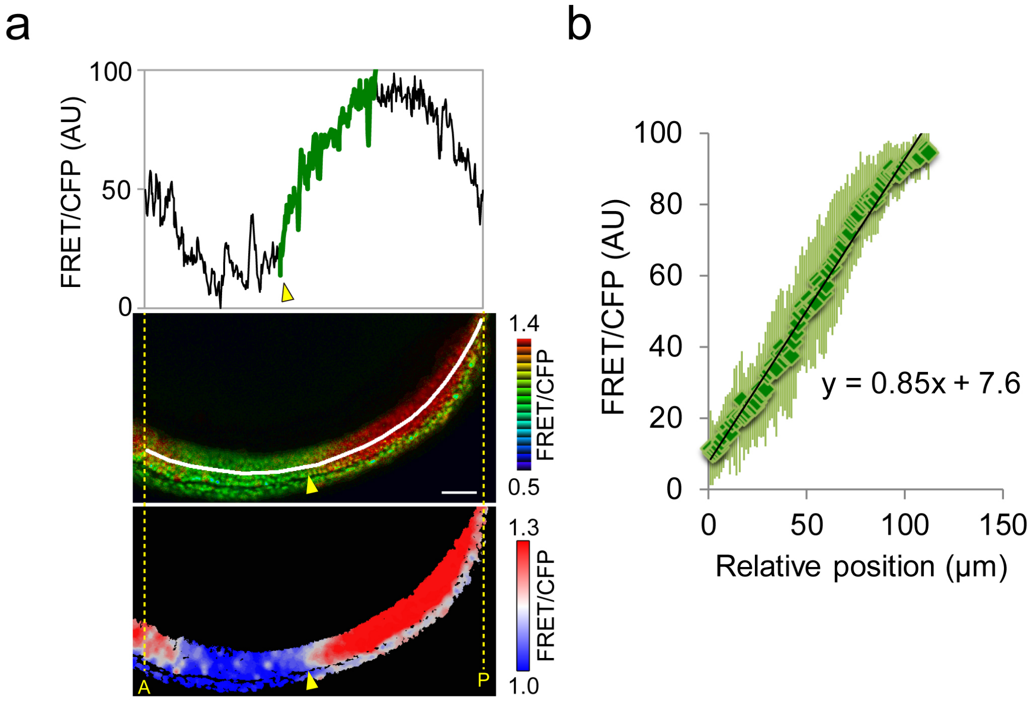

Supplementary Figure S1. Sharp border of Erk activity in control embryos.

(a) Signal intensities (upper panel) were recorded along the white curved line in somites and the entire PSM region (middle panel). The anterior extremity of Erk border, marked by yellow arrowhead in upper and middle panels, is also consistent with the position of the central value of the FRET/CFP ratio in 3-color heat map (lower panel). A and P indicate the anterior and posterior ends of the PSM, respectively. Scale bar, 50 μm. **(b)** The upward sloping region marked by the green line in panel **a** was extracted from each sample, and Erk activity slopes in control (n = 22) were calculated from averaged signal intensities using approximation formulae.

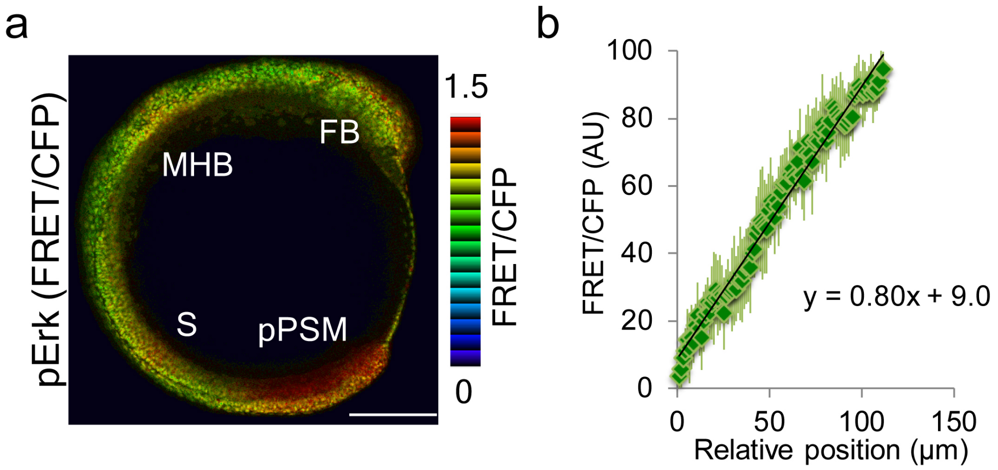

Supplementary Figure S2. A border of Erk activity is normally generated in clock-deficient embryos.

(a) Erk activity detected by the Erk biosensor in clock-deficient embryo (8-color heat map). Lateral view, anterior to the top. FB, forebrain; MHB, midbrain–hindbrain boundary; S, somite I; and pPSM, posterior PSM. Scale bar, 200 μm. **(b)** Erk activity slopes in clock-deficient embryos (n = 9) were calculated from averaged signal intensities using approximation formulae. The sharp border of Erk activity was generated in a manner similar to that of control embryos (see Supplementary Fig. S1b).

Movie legends

Movie 1. SU5402 inhibits Erk activity in the zebrafish embryo.

FRET/CFP signals were monitored at 5-min intervals. At time point 0, SU5402 was added. High FRET/CFP signals in the somite and posterior PSM gradually decreased to their basal levels after SU5402 treatment.

Movie 2. PD184352 inhibits Erk activity in the zebrafish embryo.

The FRET/CFP signals were monitored at 5-min intervals. At time point 0, PD184352 was added. High FRET/CFP signals in the somite and posterior PSM gradually decreased to their basal levels after PD184352 treatment.

Movie 3. Dynamics of the border of Erk activity in the control embryo.

The FRET/CFP signals were monitored at 5-min intervals. Left panel, 8-color heat map; right panel, 3-color heat map. White arrowheads indicate the positions of the borders of Erk activity.

Movie 4. Retrograde cell tracking in the control embryo.

Left panel, 8-color heat map; right panel, 3-color heat map. The FRET/CFP signals were monitored at 5-min intervals. At time point 02:25, anterior boundary cells of the S13 and forming somite (it will be the S14) were marked by dots (white in left panels; green in right panels), and their trajectories were retrogradely tracked. At time point 00:40, the boundary cells at the forming somite overlapped with the position of the Erk activity border (arrowheads) and the overlap was maintained until 00:35. The 5-min overlapping of the tracked cells with the Erk activity border (arrowheads) was also seen in the boundary cells at the S13 during 00:10-00:05.

Movie 5. Dynamics of the border of Erk activity in the clock-deficient embryo.

The FRET/CFP signals were monitored at 5-min intervals. Left panel, 8-color heat map;

right panel, 3-color heat map. White arrowheads indicate the positions of the borders of Erk activity.

Movie 6. Retrograde cell tracking in the clock-deficient embryo.

Left panel, 8-color heat map; right panel, 3-color heat map. The FRET/CFP signals were monitored at 5-min intervals. At time point 02:05, anterior boundary cells of the S13 and forming somite (it will be the S14) were marked by dots (white in left panels; green in right panels), and their trajectories were retrogradely tracked. The 5-min overlapping of the tracked cells with the Erk activity border (arrowheads) was seen in the boundary cells at the forming somite during 00:35-00:30 and in the cells at the S13 during 00:15-00:10.

Movie 7 Estimation of time interval between the sequential stepwise shift and size of the corresponding somite in the control embryo. Left panel, 8-color heat map; right panel, 3-color heat map. The FRET/CFP signals were monitored at 5-min intervals. Erk border cells were marked by white or green dots as shown in left or right top panels, respectively, and tracked their trajectories. Because the Erk activity border appeared at time points 00:05 and 00:35, we could estimate that time interval between the sequential stepwise shift is 30 min. At time point 02:25, we could also estimate the size of the corresponding somite (52 μm), which is generated by the sequential stepwise cycle.

Movie 8 Estimation of time interval between the sequential stepwise shift and size of the corresponding somite in the clock-deficient embryo. Left panel, 8-color heat map; right panel, 3-color heat map. The FRET/CFP signals were monitored at 5-min intervals. Erk border cells were marked by white or green dots as shown in left or right top panels, respectively, and tracked their trajectories. Because the Erk activity border appeared at time points 00:10 and 00:30, we could estimate that time interval between the sequential stepwise shift is 20 min. At time point 02:15, we could also estimate the

size of the corresponding somite (39 μm), which is generated by the sequential stepwise cycle.
